# Exogenous Application of Melatonin and Methyl Jasmonate as a Pre-Harvest Treatment Enhances Growth of Barhi Date Palm Trees, Prolongs Storability, and Maintains Quality of Their Fruits under Storage Conditions

**DOI:** 10.3390/plants11010096

**Published:** 2021-12-29

**Authors:** Waleed M. E. Fekry, Younes M. Rashad, Ibrahim A. Alaraidh, Taha Mehany

**Affiliations:** 1 Department of Plant Production, Arid Lands Cultivation Research Institute, City of Scientific Research and Technological Applications, New Borg El-Arab City 21934, Alexandria, Egypt; botanist77@yahoo.com; 2 Plant Protection and Biomolecular Diagnosis Department, Arid Lands Cultivation Research Institute, City of Scientific Research and Technological Applications (SRTA-City), New Borg El-Arab City 21934, Alexandria, Egypt; 3 Department of Botany and Microbiology, College of Science, King Saud University, Riyadh 2455, Saudi Arabia; ialaraidh@ksu.edu.sa; 4 Department of Chemistry, University of La Rioja, C/Madre de Dios 51, 26006 Logroño, La Rioja, Spain; tahamehany@yahoo.com; 5 Food Technology Department, Arid Lands Cultivation Research Institute, City of Scientific Research and Technological Applications, New Borg El-Arab City 21934, Alexandria, Egypt

**Keywords:** date palm, melatonin, methyl jasmonate, postharvest, storage

## Abstract

Fresh date palm fruits (cv. Barhi) have received much attention due to their sweet taste and popularity in marketing. There is a critical need to prolong their storability, as well as maintain their quality during the postharvest and marketing periods. In this study, the effects of spraying date palm trees with melatonin (Mt) and/or methyl jasmonate (Mj) at 10, 20, and 50 ppm, on their growth and yield were investigated. In addition, impacts on quality and storability of the fruits were also studied. In general, application of Mt was mostly more effective than that of Mj, even at 50 ppm, with regard to all evaluated parameters. However, the dual treatment at 50 ppm recorded the highest relative chlorophyll and nutrient content in date palm leaves, as well as the yield and its components. Regarding the date palm fruits stored at 4 °C for 28 days, this dual treatment recorded the lowest weight loss and fruit decay values (0.14 and 2%, respectively), the highest firmness (6 g·cm^−2^), total soluble solids content (36 °Brix), total sugar content (32.5 g/100 g fresh weight), and the lowest total acidity (0.16 g citric acid/100 mL juice). Moreover, the highest total phenolic content and activity of peroxidase and polyphenol oxidase enzymes in the stored fruits were also recorded for the dual treatment. In contrast to the untreated fruits, scanning electron microscopy observations showed that the sprayed fruits had a very good microstructure, showing intact and thick exocarp tissue with a dense layer of epicuticular wax. The mesocarp tissue showed a normal and clear cellular framework with well organized and arranged cells, after 28 days storage at 4 °C. Based on these results, we can conclude that application of the dual treatment (Mt + Mj) at 50 ppm is a promising way to prolong the storability of date palm fruits and maintain their quality during storage periods.

## 1. Introduction

Date palm (*Phoenix dactylifera* L.) is one of the most important crops cultivated in the Middle East region, especially the Arabian Peninsula and North Africa countries. Globally, Egypt is ranked first among the top date producing countries with a total production of around 1.6 million tons in 2019, while Saudi Arabia came second in this regard [1]. Date palm is gaining crucial significance in the cultural heritage of the Arab region, being one of the most ancient cultivated crops in history (3300–3100 BC) [2]. Date palm fruits have a high nutritional value, containing up to 88% carbohydrate, 5.6% protein, 11.5% fiber, as well as various minerals and vitamins. Furthermore, their seeds contain up to 58.8% of oleic acid, which makes them an economic source for oleic acid production [3]. Therefore, it may be considered as the ideal healthy food for a person. Fruits of date palm have five stages of developmental and ripening stages: Hababouk, Kimri, Khalal, Rutab, and Tamer, respectively. Usually, date palm fruits are harvested and marketed during the last three stages depending on the climatic conditions, cultivar properties, and market demand [4]. Fresh date palm fruits (cv. Barhi) have received much attention due to their sweet taste and popularity in marketing. Barhi dates are more favored and higher in marketing price at the Khalal stage compared to the Rutab and Tamer stages [5]. Therefore, there is a critical need to prolong their storability, as well as maintain their quality during the postharvest and marketing periods. 

Melatonin (Mt), *N*-acetyl-5-methoxytryptamine, is an indoleamine compound, which has been biosynthesized by various plant species. This multifunctional molecule is involved in a diverse set of physiological actions in plants including seed germination, growth regulation, photosynthesis, signaling, morphogenesis, osmoregulation, biotic and abiotic stress tolerance, delay of senescence, and acts as an antioxidant against reactive oxygen species (ROS) and nitrogen species [6]. Some recent researchers have considered plant melatonin as a new phytohormone, while others have considered it as a plant master regulator [7]. Liang et al. [8] reported that exogenous application of melatonin at 200 μmol L^−1^ resulted in a significant senescence delaying in leaves of kiwifruit seedlings by inducing the antioxidant activity and stimulating flavonoid biosynthesis. In another study, Zhong et al. [9] found that spraying grape seedlings with melatonin at 150 μmol L^−1^ promoted their growth and development, enhanced the photosynthesis in their leaves, and improved their sucrose metabolism.

Methyl jasmonate (Mj), the methyl ester of jasmonic acid, is an important phytohormone, which has many physiological and developmental roles in plants, including growth promotion, signaling, fertility, stress tolerance, senescence, seed germination, formation of storage organs, fruit ripening, stimulation of antioxidant activities, nectar biosynthesis, and regulation of various defense-related genes [10,11,12]. Fan et al. [13] reported that exogenous application of MJ at 5 mM inhibited ethylene accumulation, significantly delayed postharvest ripening and senescence in eggplant fruits, maintained their quality, and prolonged their postharvest life during storage. In contrast, Saniewski et al. [14] reported the inducing effect of Mj application on senescence in the abaxial side of *Ginkgo biloba* leaves, while little effects were observed when Mj was applied on the adaxial side of the leaves. 

This study aimed to (1) investigate the effects of spraying date palm trees with Mt and/or Mj at different concentrations on their total chlorophyll and nutrient contents, yield, and its components under field conditions and (2) study their impacts on the anatomy, quality, and storability of their fruits. 

## 2. Results

### 2.1. Effects on Relative Chlorophyll Pigments

**Figure 1:**
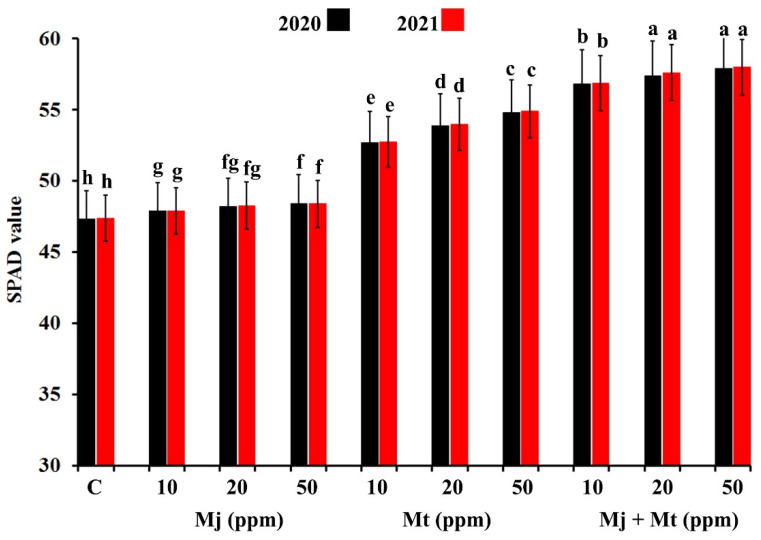
Histogram showing the relative chlorophyll content in date palm leaves (cv. Barhi) at the harvest time of two seasons (2020 and 2021) in response to spraying with methyl jasmonate and/or melatonin solutions at 10, 20, and 50 ppm. Where, C = untreated control fruits, Mj = treated with methyl jasmonate, Mt = treated with melatonin, and Mj + Mt = treated with methyl jasmonate and melatonin. For each year, columns superscripted with the same letter are not significantly different according to Tukey’s HSD test (*p* ≤ 0.05). Each value represents the mean of three replicates. Error bars represent standard errors.

Relative chlorophyll content in date palm leaves at the harvest time of two seasons (2020 and 2021), in response to spraying with Mt and/or Mj solutions at 10, 20, and 50 ppm, are illustrated in Figure 1. The obtained results showed that all applied treatments led to a significant increase in the relative chlorophyll content in date palm leaves in both seasons, compared with the untreated control leaves. In this regard, the relative chlorophyll content increased with the increment in the treatments’ concentration in both seasons. Spraying with Mt considerably enhanced the relative chlorophyll content more than Mj in both seasons. The dual treatment enhanced the relative chlorophyll content more than the individual treatments, compared with the untreated control leaves. In both seasons, the highest relative chlorophyll content was recorded for the date palm leaves treated with the dual treatment at 20 and 50 ppm, compared with the untreated control leaves.

### 2.2. Effects on the Nutrient Contents

Nutrient contents of date palm leaves, in response to foliar application of Mt and/or Mj solutions at different concentrations, are presented in Table 1. The results indicated that spraying with Mj at 50 ppm significantly enhanced the nitrogen (N) content in date palm leaves, while no change in N content was observed at 10 and 20 ppm during the first season. However, an increase in N content was recorded after spraying at 20 ppm in the next season, compared with the untreated control trees. Spraying the date palm trees with Mt at all tested concentrations considerably enhanced the N content more than spraying with Mj at 50 ppm during both seasons. Our results revealed that spraying with a mixture of Mt and Mj significantly enhanced N content more than the individual treatments during both seasons. The maximum N content was recorded for the dual treatment at 50 ppm during both seasons, compared with the untreated control treatment. Regarding phosphorus (P) content, all tested treatments during both seasons significantly led to an increase in P content, at varying extents, compared with the untreated control trees. However, P content in response to spraying with Mt at any tested concentration was more than that due to spraying with Mj, even at its highest tested concentration. At the same concentration, spraying with the dual treatment increased P content more than the individual treatments during both seasons. The highest P content was recorded for the dual treatment at 20 and 50 ppm during both seasons, compared with the untreated control trees. For potassium (K) content, the obtained data indicated that spraying date palm trees with Mj at 50 ppm increased the K content, while no change was observed after spraying with Mj at 10 and 20 ppm during both seasons, when compared with the untreated control trees. During both seasons, spraying with Mt at any tested concentrations increased the K content more than that of Mj, even at its highest tested concentration. At the same concentration, spraying with the dual treatments enhanced the K content more than the individual ones during both seasons. The highest K content was recorded for date palm trees sprayed with the dual treatment at 50 ppm during both seasons, compared with the untreated control trees. 

### 2.3. Effects on the Yield and Its Components

Effects on the yield and its components, in response to foliar application of Mt and/or Mj solutions at different concentrations, are shown in Table 2. The obtained results indicated that all tested treatments significantly enhanced the yield/palm during both seasons, compared with the untreated control trees. Spraying with Mt at any tested concentration increased the yield/palm more than that with Mj, even at 50 ppm. At any concentration, the dual treatment enhanced the yield/palm more than the individual treatments by around 45% during both seasons. During both seasons, the highest yield/palm value was recorded for the dual treatment at 50 ppm, compared with the untreated control trees. Regarding the bunch weight, spraying date palm trees with Mj at 20 and 50 ppm considerably increased the bunch weight, while no change was detected at 10 ppm during both seasons, compared with the untreated control trees. During both seasons, all applied treatments of Mt led to an enhancement in the bunch weight higher than that of Mj by around 18%. Except at 10 ppm during the first season, the dual treatments enhanced the bunch weight more than the individual treatments by around 35%, compared with the untreated control trees. The highest bunch weight value was recorded for the dual treatment at 50 ppm during both seasons, compared with the untreated control trees. For fruit length, no change was observed for the foliar application of Mj at any concentration during both seasons, compared with the untreated control. In contrast, all tested concentrations of Mt enhanced the fruit length during both seasons, compared to the untreated control trees. During both seasons, the dual treatments were more efficient than the individual treatments, in this regard, by around 16%. The highest fruit length value was recorded for the dual treatment at 50 ppm during both seasons, when compared with the untreated trees. Regarding fruit diameter, spraying with Mj at 50 ppm increased the fruit diameter during both seasons, while no change was observed at 10 and 20 ppm, when compared with the untreated control treatment. During both seasons, foliar application of Mt significantly improved the fruit diameter more than treatment of Mj at 50 ppm by ≈ 6%, compared with the untreated control trees. During both seasons, the dual treatment enhanced the fruit diameter higher than the individual ones. Compared with the untreated trees, the highest fruit diameter value was recorded for the dual treatment at 50 ppm during both seasons.

### 2.4. Effect on the Fruit Weight Loss

Mean weight loss percentage in date palm fruits during storage at 4 °C for 28 days, in response to foliar application of Mt and/or Mj solutions at different concentrations, are shown in Table 3. The weight loss percentage increased in the date palm fruits with the increment in storage time for all treatments in both seasons. At 14 days post-harvest (dph), date palm fruits treated with Mt and/or Mj showed a significant reduction in the weight loss percentage in both seasons, compared with the untreated control fruits. However, the dual treatment mostly reduced the weight loss percentage more than the individual ones by ≈60%. Increase in concentration of the tested treatments led to more reduction in the weight loss percentage, compared with the untreated control fruits. At 28 dph, the weight loss percentage in date palm fruits increased compared to that at 14 dph in both seasons. In both seasons, all applied treatments considerably reduced the weight loss, compared with the untreated control fruits. The lowest weight loss percentage values were recorded for the date palm fruits treated with the dual treatment at 50 ppm during both seasons recording 0.16 and 0.14 %, respectively.

### 2.5. Effect on the Fruit Decay

Mean fruit decay in date palm fruits during storage at 4 °C for 28 days, in response to foliar application of Mt and/or Mj solutions at different concentrations, are shown in Table 3. The fruit decay increased with the increment in the storage period for all treatments, at varying degrees. After 14 dph, all tested treatments significantly reduced the fruit decay in stored date palm fruits, compared with the untreated fruits during both seasons. In this regard, the fruit decay, due to the dual treatment, was mostly less than the individual ones by ≈75% during both seasons. The lowest fruit decay was recorded for the date palm fruits sprayed with the dual treatment at 50 ppm during both seasons, when compared with the untreated control fruits. After 28 dph, the higher fruit decay value was recorded for the untreated fruits during both seasons recording 15 and 15.7%, respectively. Spraying with all tested treatments considerably reduced the fruit decay during both seasons. The lowest fruit decay was recorded for the fruits treated with the dual treatment at 50 ppm at 28 dph, recording 2% during both seasons. 

### 2.6. Effect on the Fruit Firmness

Mean firmness of date palm fruits during storage at 4 °C for 28 days, in response to spraying with Mt and/or Mj solutions at different concentrations, are showed in Table 4. The obtained results indicated that the fruit firmness decreased with the increment in the storage period. All tested treatments significantly enhanced the fruit firmness during the storage period in both seasons, when compared with the untreated control fruits. During both seasons, the increase in concentration of the applied treatments led to a considerable increase in the fruit firmness. In both seasons, none of the treatments affected the fruit firmness at harvest compared to the control fruits, which corresponded to 6.6 and 6.3 g cm^−2^ in 2020 and 2021, respectively. During both seasons, spraying date palm fruits with Mt mostly increased their firmness more than Mj, especially at the highest concentration. However, the dual treatment was found more effective in increasing the fruit firmness at both 14 and 28 dph, in both seasons.

### 2.7. Effect on the Total Soluble Solids Content (TSS)

Mean TSS content in date palm fruits during storage at 4 °C for 28 days, in response to spraying with Mt and/or Mj solutions at different concentrations, are presented in Table 5. The obtained results showed that TSS content increased with increment in the storage period for treated and untreated date palm fruits. At harvest time, all tested treatments significantly enhanced the TSS content in date palm fruits during both seasons, compared with the untreated control fruits. Spraying date palm fruits with Mt increased TSS more than Mj during both seasons. The dual treatment mostly increased TSS content higher than the individual treatments during both seasons, compared to the untreated control. The highest TSS content was recorded for the dual treatment at 50 ppm at 28 dph recording 36 °Brix for each season. 

### 2.8. Effect on the Total Sugars Content (TS)

Mean TS in date palm fruits during storage at 4 °C for 28 days, in response to spraying with Mt and/or Mj solutions at different concentrations, are presented in Table 6. The obtained data showed that TS content increased at 14 dph more than that at the harvest time. whereas it reduced again at 28 dph in all treatments during both seasons. Both Mt and Mj treatments increased the TS content compared to controls in both seasons, with a positive relationship with the increase in their concentrations. In general, Mt treatments were more effective in improving the TS content than Mj treatments by ≈10 %. Furthermore, the dual treatment mostly enhanced the TS content more than the individual ones by 15% during both seasons.

### 2.9. Effect on the Total Acidity (TA)

Mean TA in date palm fruits during storage at 4 °C for 28 days, in response to spraying with Mt and/or Mj solutions at different concentrations, are presented in Table 7. The results indicated that TA in date palm fruits reduced with the increment in the storage period for both treated and untreated fruits with varying degrees. All tested treatments significantly reduced the TA in the treated fruits at varying degrees, compared with the untreated control fruits. At harvest time, spraying with Mt at all tested concentrations reduced the TA more than with Mj, even at its highest concentration during both seasons. The dual treatment mostly reduced the TA more than the individual ones and Mt treatment was more effective than Mj treatment by ≈20% during both seasons, compared with the untreated control fruits. The lowest TA was recorded for the dual treatment at 20 and 50 ppm at 28 dph in each season.

### 2.10. Effect on the Total Phenolic Content and Activity of Peroxidase and Polyphenol Oxidase Enzymes

**Figure 2:**
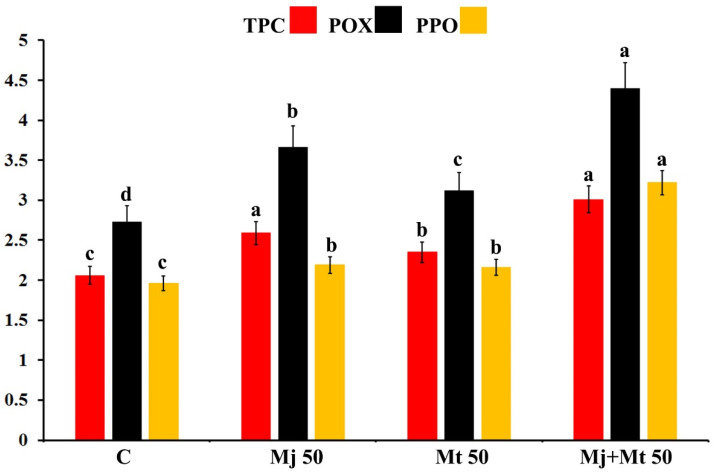
Histogram showing the total phenolic content (TPC, mg gallic acid equivalent/g fresh weight), and activity of peroxidase (POX, ∆_A470_ min^−1^ g^−1^ fresh weight) and polyphenol oxidase (PPO, ∆A_420_ min^−1^ g^−1^ fresh weight) enzymes in date palm fruits (cv. Barhi) after 28 days of storage at 4 °C, in response to spraying with methyl jasmonate and/or melatonin solutions at 50 ppm. Where, C = untreated control fruits, Mj 50 = treated with methyl jasmonate at 50 ppm, Mt 50 = treated with melatonin at 50 ppm, and Mj + Mt 50 = treated with methyl jasmonate and melatonin at 50 ppm. For each parameter, columns superscripted with the same letter are not significantly different according to Tukey’s HSD test (*p* ≤ 0.05). Each value represents the mean of three replicates. Error bars represent standard errors.

Mean total phenolic content (TPC) and activity of peroxidase (POX) and polyphenol oxidase (PPO) enzymes in date palm fruits after 28 days of storage at 4 °C, in response to spraying with Mj and/or Mt at 50 ppm, are illustrated in Figure 2. The obtained data revealed that spraying the date palm fruits with Mj and/or Mt significantly increased the TPC, compared with the untreated control treatment. In this regard, spraying with Mj alone or with Mt at 50 ppm enhanced the TPC more than Mt alone at 50 ppm, compared with the untreated control treatment. Regarding POX, all applied treatments led to a considerable increase in the enzyme activity, compared with the untreated control treatment. For both POX and PPO, the dual treatments were more effective in enhancing the activity of the enzymes, compared to the individual treatments.

### 2.11. Correlations between the Recorded Variables from the Date Palm Trees

Correlation values between the recorded variables from the date palm trees are presented in Table 8. The obtained results revealed that most of the evaluated variables (relative chlorophyll content, nitrogen content, phosphorus content, potassium content, yield/palm, bunch weight, fruit length, fruit diameter, total soluble solids content, total sugars, and fruit firmness) had strong positive correlations with each other (r = 0.37–0.99). In contrast, total acidity, fruit weight loss, and fruit firmness were negatively correlated with the other variables (r = −0.37–0.97), and positively with each other (r = 0.38–0.87).

### 2.12. Scanning Electron Microscopy (SEM) 

**Figure 3:**
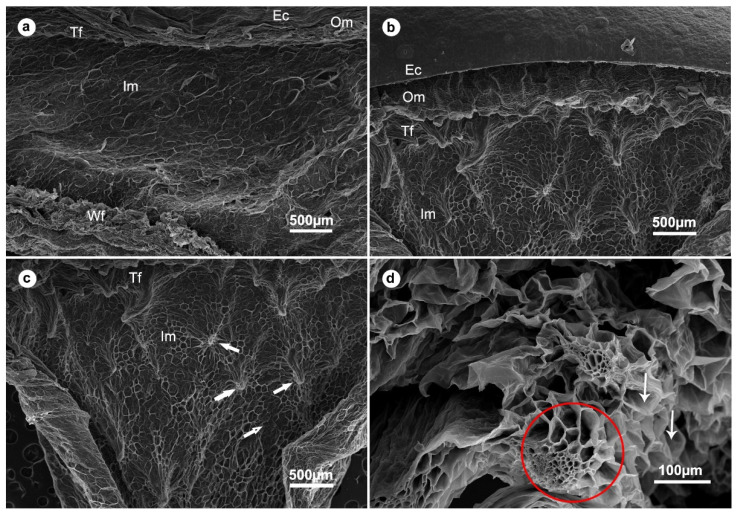
Scanning electron micrographs of date fruit showing effect of spraying with methyl jasmonate and melatonin at 50 ppm on the fruit tissues. Where (**a**) untreated fruit (control) showing the exocarp (Ec), which is comprised of layers of the cuticle, epidermis, hypodermis, and sclereid cells, the outer mesocarp (Om), tanniferous layer (Tf), inner mesocarp (Im), and white fiber bundles (Wf), (**b**) treated fruit showing Ec, Om, Tf, Im layers, (**c**) a micrograph focusing on the inner mesocarp (Im) of the treated fruit showing the fibrovascular bundles (thick arrows), and (**d**) an enlarged micrograph of treated date fruit showing vascular bundles (red circle) with xylem vessels and sieve tubes. The thin arrows show the lumen of each cell.

The effect of foliar application of Mt and Mj at 50 ppm on the microstructure of date fruits stored at 4 °C for 28 days was investigated using SEM (Figure 3). SEM observations of the unsprayed fruit showed that all fruit tissues appeared to have shrunk, especially the exocarp tissue, which is composed of four layers of cuticle, epidermis, hypodermis, and sclereid cells. The mesocarp and tanniferous layers showed a thin cellular framework and appeared in disorganized arrangement, while the white fiber bundles were compressed and shrunk (Figure 3a). In contrast, SEM observations of the treated fruit showed an intact and thick exocarp tissue with a dense layer of epicuticular wax (Figure 3b). The mesocarp and tanniferous layers showed a normal and clear cellular framework. Their cells were well organized, arranged in an orderly and tight manner, with scattered fibro-vascular bundles (Figure 3c). Furthermore, vascular bundles consisting of xylem vessels and sieve tubes appeared intact, with a clear cellular structure and lumen (Figure 3d). 

## 3. Discussion

Date palm fruits (cv. Barhi) have received much attention due to their sweet taste and popularity in marketing. In order to prolong their shelf-life, we investigated the effects of spraying date palm trees with Mt and/or Mj at 10, 20, and 50 ppm on storability and quality of their fruits during 28 days of storage at 4 °C. Results from the field experiment showed that the highest relative chlorophyll content in date palm leaves was recorded for the date palm leaves treated with the dual treatment at 20 and 50 ppm. This result is in accordance with that obtained by Zhang et al. [15] who found that foliar spraying of ryegrass plants with Mt led to a significant enhancement in the chlorophyll content and improved the photosynthesis capacity in their leaves. The discussed mechanisms include antioxidant protection against ROS, which attack the photosynthetic apparatus including chlorophyll and the photosynthetic proteins, upregulation of the chlorophyllase gene expression, and increment of the open reaction centers of photosystem II, which led to an improvement in the net photosynthesis efficiency [16,17]. On the other hand, Sirhindi et al. [18] reported the enhancing effect of Mj application on the photosynthetic efficiency of cabbage plants. They found that the maximum quantum efficiency and stability of the photosystem II was increased and the transcriptional expression of *Psbl* gene, which has an electron transport function, was upregulated in response to the exogenous application of Mj. These led to an improvement in the efficiency and productivity of the photosynthetic apparatus of cabbage plants. This may explain the synergistic enhancing effect of the dual treatment, which we obtained in our study on the relative chlorophyll content in date palm leaves. 

Results obtained from this study revealed that the dual treatment enhanced the yield and its components in date palm trees. Various recent studies reported Mt as a master regulator in plants promoting vegetative growth, seed germination, flowering, and fruiting in many plant species [19]. The growth promoting effect of Mt has been discussed in the light of its inducing effect on the auxin-signaling pathway and regulating transcriptional expression of many auxin related transcriptional factors [20]. Furthermore, Mt triggers metabolism of most phytohormones, such as indole acetic acid, abscisic acid, cytokinins, and gibberellic acid [21]. On the other hand, application of Mj has been reported to enhance yield and fruit quality in pomegranate trees [22]. The yield increase is due to the increment in the number of harvested fruits, and content of the photosynthetic pigments [18]. Mj is a natural plant-signaling molecule, which has various eliciting effects on the plant growth on cellular and molecular levels via regulation of multiple growth-related genes expression [23]. 

**Figure 4:**
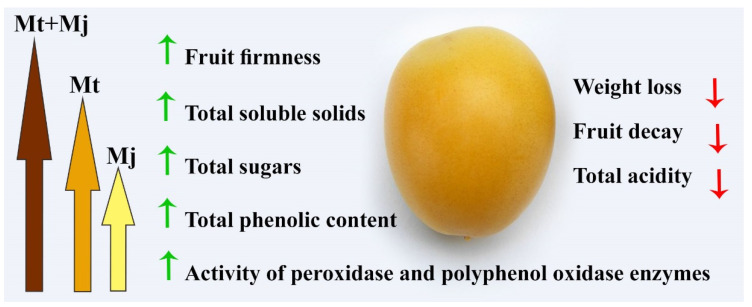
Schematic figure illustrating the main results obtained in this study. Where, Mj = methyl jasmonate, and Mt = melatonin.

One of the interesting results obtained in this study is the reducing effect of the dual treatment on the weight loss and fruit decay in date palm fruits stored for 28 days. Recent research has reported the defense mechanisms of Mt, such as ROS scavenging by inducing the antioxidant enzymes, such as peroxidase, catalase, superoxidedismutase, polyphenoloxidase, and glutathione reductase, as well as eliciting expression of their encoding genes under different abiotic and biotic stress conditions [24]. Furthermore, non-enzymatic antioxidants, such as phenolics and carotenoids, are also triggered by Mt application [25]. This information is confirmed by the enhancement in the total phenolic content and activities of POX and PPO enzymes, which was recorded in our study in date palm fruits in response to the Mt treatments. Chilling damage during storage was found to be associated with elevated ethylene production and respiration rate [26]. In this concern, Zhai et al. [27] reported that Mt application led to a reduction in ethylene production and respiration rates in pears, resulting in a delay in their senescence. Inhibition of ethylene burst in fruits treated with Mt leads to enhancement of its firmness. The obtained results in this study showed the Mt application significantly enhanced the fruit firmness. Downregulation of *PcPG*, a main cell-wall degradation gene, in response to Mt application has also been reported as another anti-senescence and storage quality maintenance mechanism. Serrano et al. [28] studied the physicochemical alterations during the ripening stages of date palm fruits and found that the highest reduction in fruit firmness was associated with the increase in the enzymatic activity of polygalacturonase and *β*-galactosidase. These enzymes and expansin (SlEXP1) proteins are mainly responsible for fruit softening during ripening via disassembling of the cell wall polysaccharide network, which represents 90% of the cell wall composition [29]. In this regard, Tang et al. [30] found that Mt treatment delayed softening of jujube fruits by suppressing activity of the cell wall-degrading enzymes pectin methylesterase, polygalacturonase, cellulose, and *β*-glucosidase, as well as enhancing water soluble pectin accumulation in addition to the water insoluble pectin. This may discuss the firmness of fruit tissues, especially the cuticle layer observed by SEM in the treated date palm fruits in this study. On the other hand, Baswal et al. [31] reported that the application of Mj on mandarin fruit led to an inhibition in ethylene content and induced contents of total phenols, flavonoids, free amino acids, sugars, and total antioxidants. This result is in agreement with that obtained in this study on date palm fruits. The same results were obtained by Boonyaritthongchai and Supapvanich [32] on pineapples. The immersing of pineapples in Mj (1 mM) was found to inhibit ethylene production, weight loss, internal browning, and enhance the fruit firmness during 20 days of storage at 10 °C. Moreover, this treatment led to an induction of the antioxidant machinery system via enhancing the total phenols content and the enzymatic activity of PPO and superoxide dismutase to reduce cellular levels of H_2_O_2_ and ROS. In another recent study, it was demonstrated that exogenous application of Mj delayed the postharvest physiological deterioration and cell oxidative damage in cassava and induced transcriptional expression of Mt biosynthesis genes [33]. Elevation of the endogenous Mt content in response to the exogenous application of Mj may discuss the synergistic effect of the dual treatment tested in our study and its superiority over the individual treatments. Figure 4 summarizes the main results obtained in this study on date fruits. Based on these results, we concluded that application of the dual treatment (Mt + Mj) is a promising way to prolong the storability of date palm fruits and maintain their quality during storage periods.

## 4. Materials and Methods

### 4.1. Chemicals and Date Palm Cultivar

The chemical substances used in this study (Mj and Mt) were purchased from Sigma-Aldrich (St Louis, MO, USA). Date palms trees of Barhi cultivar were used to conduct the field experiment.

### 4.2. Field Experiment

The field experiment was performed at a date palm farm located in Mandisha village, Baharia oasis, Giza, Egypt. Thirty date palm trees (cv. Barhi) of uniform vigor, 12-years-old, free of diseases and insect damages, and grown in sandy soil under a drip irrigation system were selected. The soil physicochemical properties were as follows: sandy texture, organic matter (0.5%), pH (7.9), electrical conductivity (1.6 dSm^−1^), calcium content (3.5%), total N content (0.08%), available P content (2.5 ppm), and available K content (75 ppm). The date palm trees were spaced at 8 × 8m and all trees received the same horticulture practices as recommended by the Ministry of Agriculture. At the beginning of each season, the number of inflorescences was adjusted to 10 per tree. Bunches of each date palm tree were thinned to the same number of strands. All trees uniformly received the same artificial pollination practices. The date palm trees were separately sprayed with a solution of Mt at 10, 20, and 50 ppm and/or MJ at 10, 20, and 50 ppm (10 L/tree) two times at 2 months pre-harvest (first of July), and 1 month pre-harvest (first of August). Date palm trees sprayed with water were used as the control treatment. The experiment included ten treatments arranged in a randomized complete block design. Each treatment was performed in triplicate, one tree per block. The experiment was repeated over 2 seasons during July–September 2020 and 2021. During the experiment period, the weather conditions were as follows: average temperature (22–38 °C), relative humidity (57–66%), rainfall (0–0.4 mm), and daily sun hours (11.8–12 h).

#### 4.2.1. Biochemical Analyses of Date Palm Leaves

For each season at the harvest time, date palm leaves from each treatment were analyzed for the relative chlorophyll content (chlorophyll *a*, and *b*) using a SPAD-502 chlorophyll meter (Konica-Minolta, Tokyo, Japan). In addition, N content was estimated in date palm leaves by the modified Kjeldahl method [34]. Total P content was determined using the spectrophotometric vanadium phosphomolybdate method [35] using a spectrophotometer (Unico, Model UV2150, Dayton, NJ, USA), and the total K content was estimated according to Cottenie et al. [36] using a flame photometer (Systonic, Model S-935, Haryana, India). For each treatment three replicates were applied.

#### 4.2.2. Yield and Its Components

For each season at the harvest time, ten bunches per tree were weighted to determine the average bunch weight (kg) and the average yield per palm (kg). In addition, fruit length and diameter (cm) were also measured using a Vernier caliper.

#### 4.2.3. Postharvest Physical Properties of Date Palm Fruits

For each season at the harvest time, fifty date palm fruits from each treatment were washed with tap water, air dried, weighed to determine the average fruit weight at the harvest time, and stored in a refrigerator at 4 °C for 28 days. The average fruit weight was determined again 14 and 28 dph to calculate the weight loss of fruits (%) using the following equation:

$$\mathrm{Fruit}\;\mathrm{weight}\;\mathrm{loss}\;\left(\%\right)=\frac{\mathrm{Initial}\;\mathrm{weight}-\mathrm{weight}\;\mathrm{at}\;\mathrm{specific}\;\mathrm{time}}{\mathrm{Initial}\;\mathrm{weight}}\times 100$$

In addition, the fruit decay and firmness were also determined for the date palm fruits at harvest time, 14 dph, and 28 dph. To determine the fruit decay, the fruits were checked for skin appearance, shriveling, injury, and pathogenic rots. The decayed fruits from each treatment sample were discarded and weighed. The fruit decay (%) was calculated using the following equation:

$$\mathrm{Fruit}\;\mathrm{Decay}\;\left(\%\right)=\frac{\mathrm{Weight}\;\mathrm{of}\;\mathrm{discarded}\;\mathrm{fruits}}{\mathrm{Initial}\;\mathrm{fruit}\;\mathrm{weight}}\times 100$$

Firmness of the date palm fruits (g.cm^−2^) was determined using a fruit pressure tester (Penetrometer ST 308, Milan, Italy).

#### 4.2.4. Postharvest Chemical Properties of Date Palm Fruits

For each season at the harvest time, 14 dph, and 28 dph, date palm fruits from each treatment were analyzed for TSS (°Brix) using a hand refractometer model Master T (ATAGO Co., Ltd., Tokyo, Japan). TS content (g/100 g fresh weight) was determined according to the method of Sadasivam and Manickam [37]. TA (g citric acid/100 mL juice) was determined as described by AOAC [38].

#### 4.2.5. Biochemical Analyses of Date Palm Fruits

Twenty-eight days post-harvest, date palm fruits from each treatment were analyzed for TPC according to Malick and Singh [39]. A sample of the date palm fruit (1 g) was ground in 10 mL ethanol (80%), and centrifuged at 5000 rpm for 15 min. The supernatant was collected and the residue was re-extracted with 5 mL ethanol (80%), and re-centrifuged. The supernatant was collected together and evaporated to dryness. Five milliliters of distilled water were added to the residue. A known volume (0.2 mL) was pipetted into a clean test tube. The volume was made up to 3 mL with distilled water, to which 0.5 mL of folin-Ciocalteau reagent was added. After 3 min, 2 mL of 20% Na_2_CO_3_ solution were added. The contents were mixed thoroughly and placed in a boiling water bath for 1 min, cooled, and the absorbance was measured at 650 nm against blank. 

For the enzyme crude extract preparation, one gram of the date fruit tissue was ground with 2 mL K-phosphate buffer (0.2 M, pH 7.0). The homogenate was centrifuged at 5000 rpm for 15 min at −4 °C. Using the same buffer, the supernatant was made up to a known volume. Activity of POX and PPO enzymes were spectrophotometrically estimated according to the methods adopted by Maxwell and Bateman [40], and Galeazzi et al. [41], respectively.

#### 4.2.6. SEM Observations

A piece (1 cm^2^) of the date palm fruit treated with Mj + Mt at 50 ppm and an untreated one were dehydrated using 80% ethanol five times for 10 min, then, using acetone by washing twice, coated with gold using a sputter coater (FDU-010). SEM observations were performed using a scanning electron microscope (JEOL, 100 CX-II ASID-4D, Tokyo, Japan). 

### 4.3. Statistical Analyses

Statistical significances were analyzed using the software CoStat, version 6.4, (CoHort software, Monterey, CA, USA). The analyzed data were first examined for normality and then subjected to analysis of variance. Comparisons between the means were performed using Tukey’s HSD test at *p* ≤ 0.05 based on one-way ANOVA. Correlations among variables were analyzed using R Core Team-version 4.1.0 [42].

## Figures and Tables

**Table 1:** Effects of foliar application of melatonin and/or methyl jasmonate solutions at different concentrations on nutrient contents of date palm leaves (cv. Barhi) at the harvest time *.

Treatment		Nitrogen (%)		Phosphorus (%)		Potassium (%)	
		2020	2021	2020	2021	2020	2021
Control		1.27 ± 0.02 ^g^	1.28 ± 0.01 ^g^	0.20 ± 0.01 ^g^	0.21 ± 0.01 ^g^	1.40 ± 0.01 ^f^	1.41 ± 0.02 ^f^
Methyl Jasmonate (ppm)	10	1.27 ± 0.04 ^g^	1.28 ± 0.01 ^g^	0.23 ± 0.01 ^f^	0.24 ± 0.01 ^f^	1.41 ± 0.01 ^ef^	1.43 ± 0.01 ^f^
	20	1.32 ± 0.02 ^fg^	1.33 ± 0.01 ^f^	0.24 ± 0.01 ^f^	0.25 ± 0.02 ^f^	1.42 ± 0.01 ^ef^	1.43 ± 0.02 ^f^
	50	1.33 ± 0.02 ^f^	1.35 ± 0.02 ^f^	0.28 ± 0.02 ^e^	0.29 ± 0.03 ^e^	1.43 ± 0.02 ^e^	1.48 ± 0.02 ^e^
Melatonin (ppm)	10	1.42 ± 0.03 ^e^	1.44 ± 0.02 ^e^	0.39 ± 0.01 ^d^	0.40 ± 0.03 ^d^	1.54 ± 0.02 ^d^	1.56 ± 0.03 ^d^
	20	1.46 ± 0.01 ^de^	1.47 ± 0.01 ^d^	0.40 ± 0.01 ^cd^	0.41 ± 0.02 ^cd^	1.55 ± 0.01 ^d^	1.58 ± 0.02 ^d^
	50	1.48 ± 0.01 ^d^	1.49 ± 0.04 ^d^	0.41 ± 0.02 ^c^	0.43 ± 0.02 ^c^	1.61 ± 0.01 ^c^	1.62 ± 0.01 ^c^
Methyl Jasmonate + Melatonin (ppm)	10	1.55 ± 0.02 ^c^	1.56 ± 0.03 ^c^	0.44 ± 0.01 ^b^	0.46 ± 0.01 ^b^	1.68 ± 0.02 ^b^	1.68 ± 0.03 ^b^
	20	1.64 ± 0.01 ^b^	1.67 ± 0.02 ^b^	0.47 ± 0.02 ^a^	0.49 ± 0.03 ^a^	1.69 ± 0.03 ^b^	1.71 ± 0.02 ^b^
	50	1.80 ± 0.03 ^a^	1.82 ± 0.03 ^a^	0.48 ± 0.02 ^a^	0.49 ± 0.03 ^a^	1.73 ± 0.03 ^a^	1.74 ± 0.03 ^a^

* In each column, values followed by the same letter are not significantly different according to Tukey’s HSD test (*p* ≤ 0.05); each value represents the mean of three replicates ± SD.

**Table 2:** Effects of foliar application of melatonin and/or methyl jasmonate solutions at different concentrations on the yield and its components of date palm trees (cv. Barhi) at the harvest time *.

Treatment		Yield/Palm (kg)		Bunch Weight (kg)		Fruit Length (cm)		Fruit Diameter (cm)	
		2020	2021	2020	2021	2020	2021	2020	2021
Control		96.4 ± 1.85 ^h^	96.6 ± 1.93 ^i^	9.47 ± 0.45 ^g^	9.57 ± 0.881 ^g^	2.99 ± 0.41 ^g^	3.00 ± 0.62 ^g^	2.52 ± 0.35 ^h^	2.53 ± 0.21 ^h^
Methyl Jasmonate (ppm)	10	100.4 ± 2.86 ^g^	100.5 ± 2.11 ^h^	9.73 ± 0.81 ^fg^	9.80 ± 1.01 ^fg^	3.01 ± 0.33 ^fg^	3.05 ± 0.81 ^fg^	2.53 ± 0.84 ^gh^	2.55 ± 0.47 ^h^
	20	104.9 ± 1.81 ^f^	105.4 ± 1.89 ^g^	10.4 ± 0.74 ^ef^	10.5 ± 0.96 ^e^	3.04 ± 0.72 ^fg^	3.07 ± 0.56 ^fg^	2.55 ± 0.32 ^gh^	2.57 ± 0.73 ^gh^
	50	105.5 ± 2.42 ^f^	105.6 ± 1.74 ^g^	10.7 ± 0.70 ^e^	10.8 ± 0.93 ^e^	3.05 ± 0.33 ^fg^	3.08 ± 0.61 ^fg^	2.57 ± 0.44 ^f^	2.59 ± 0.55 ^g^
Melatonin (ppm)	10	115.1 ± 1.78 ^e^	115.7 ± 2.01 ^f^	11.8 ± 0.91 ^d^	12.4 ± 1.13 ^d^	3.23 ± 0.64 ^e^	3.25 ± 0.47 ^e^	2.64 ± 0.62 ^e^	2.65 ± 0.51 ^f^
	20	119.1 ± 1.65 ^d^	119.3 ± 1.68 ^e^	12.2 ± 1.01 ^cd^	12.5 ± 1.12 ^d^	3.30 ± 0.44 ^d^	3.34 ± 0.63 ^d^	2.67 ± 0.94 ^e^	2.68 ± 0.32 ^e^
	50	121.3 ± 2.31 ^d^	123.0 ± 1.77 ^d^	12.7 ± 1.00 ^c^	13.0 ± 1.22 ^cd^	3.38 ± 0.95 ^c^	3.40 ± 0.70 ^c^	2.74 ± 0.32 ^d^	2.75 ± 0.19 ^d^
Methyl Jasmonate + Melatonin (ppm)	10	128.3 ± 2.57 ^c^	129.6 ± 2.10 ^c^	12.9 ± 0.98 ^c^	13.5 ± 0.99 ^bc^	3.45 ± 0.55 ^b^	3.48 ± 0.45 ^b^	2.78 ± 0.61 ^c^	2.79 ± 0.75 ^c^
	20	132.9 ± 2.30 ^b^	134.7 ± 2.11 ^b^	13.7 ± 1.02 ^b^	13.9 ± 1.30 ^b^	3.48 ± 0.43 ^b^	3.49 ± 0.69 ^b^	2.82 ± 0.49 ^b^	2.84 ± 0.84 ^b^
	50	145.5 ± 1.95 ^a^	146.3 ± 2.05 ^a^	14.3 ± 1.10^a^	14.6 ± 1.15 ^a^	3.54 ± 0.87 ^a^	3.59 ± 0.83 ^a^	2.85 ± 0.63 ^a^	2.88 ± 0.90 ^a^

* In each column, values followed by the same letter are not significantly different according to Tukey’s HSD test (*p* ≤ 0.05); each value represents the mean of three replicates ± SD.

**Table 3:** Effects of foliar application of melatonin and/or methyl jasmonate solutions at different concentrations on the weight loss (%) and the fruit decay (%) in date palm fruits (cv. Barhi) stored at 4 °C for 28 days *.

Treatment		Days after Harvest							
		Weight Loss (%)				Fruit Decay (%)			
		14	28	14	28	14	28	14	28
		2020		2021		2020		2021	
Control		1.40 ± 0.31 ^a^	3.03 ± 0.51 ^a^	1.83 ± 0.44 ^a^	3.30 ± 0.60 ^a^	6.0 ± 1.0 ^a^	15.0 ± 1.1 ^a^	6.3 ± 1.0 ^a^	15.7 ± 1.2 ^a^
Methyl Jasmonate (ppm)	10	1.04 ± 0.22 ^b^	2.27 ± 0.34 ^b^	0.93 ± 0.12 ^b^	1.67 ± 0.47 ^b^	3.0 ± 0.9 ^b^	8.67 ± 0.9 ^b^	4.0 ± 0.7 ^b^	8.67 ± 0.9 ^b^
	20	0.48 ± 0.08 ^c^	1.10 ± 0.37 ^c^	0.41 ± 0.09 ^c^	0.83 ± 0.14 ^c^	2.7 ± 0.8 ^bc^	7.67 ± 0.6 ^c^	3.7 ± 0.5 ^bc^	8.33 ± 0.8 ^bc^
	50	0.16 ± 0.03 ^d^	0.49 ± 0.09 ^d^	0.22 ± 0.05 ^d^	0.60 ± 0.11 ^d^	2.7 ± 0.7 ^bc^	6.67 ± 0.6 ^d^	3.0 ± 0.5 ^d^	8.00 ± 0.4 ^c^
Melatonin (ppm)	10	0.23 ± 0.06 ^bc^	0.49 ± 0.07 ^d^	0.20 ± 0.05 ^d^	0.49 ± 0.09 ^de^	2.3 ± 0.6 ^c^	6.33 ± 0.7 ^de^	3.0 ± 0.6 ^d^	7.33 ± 0.7 ^d^
	20	0.25 ± 0.05 ^bc^	0.42 ± 0.06 ^de^	0.18 ± 0.04 ^de^	0.42 ± 0.09 ^e^	2.3 ± 0.6 ^c^	6.33 ± 0.5 ^de^	2.7 ± 0.7 ^de^	6.67 ± 0.7 ^de^
	50	0.19 ± 0.08 ^d^	0.32 ± 0.11 ^f^	0.17 ± 0.05 ^de^	0.32 ± 0.14 ^f^	2.0 ± 0.5 ^cd^	4.67 ± 0.7 ^f^	2.7 ± 0.8 ^de^	5.00 ± 0.5 ^f^
Methyl Jasmonate + Melatonin (ppm)	10	0.16 ± 0.07 ^de^	0.31 ± 0.13 ^f^	0.16 ± 0.07 ^e^	0.31 ± 0.15 ^f^	2.0 ± 0.7 ^cd^	4.00 ± 0.5 ^fg^	2.3 ± 0.7 ^e^	5.00 ± 0.4 ^f^
	20	0.14 ± 0.05 ^e^	0.27 ± 0.08 ^g^	0.14 ± 0.03 ^e^	0.25 ± 0.09 ^g^	1.3 ± 0.5 ^e^	2.67 ± 0.6 ^h^	1.7 ± 0.3 ^f^	3.33 ± 0.6 ^g^
	50	0.10 ± 0.04 ^f^	0.16 ± 0.07 ^h^	0.11 ± 0.05 ^f^	0.14 ± 0.05 ^h^	1.0 ± 0.4 ^e^	2.00 ± 0.4 ^i^	1.0 ± 0.4 ^g^	2.00 ± 0.5 ^h^

* In each column, values followed by the same letter are not significantly different according to Tukey’s HSD test (*p* ≤ 0.05); each value represents the mean of three replicates ± SD.

**Table 4:** Effects of foliar application of melatonin and/or methyl jasmonate solutions at different concentrations on the firmness (g cm^−2^) in date palm fruits (cv. Barhi) stored at 4 °C for 28 days *.

Treatment		Days after Harvest			
		14	28	14	28
		2020		2021	
Control		5.4 ± 0.20 ^d^	4.4 ± 0.11 ^e^	5.2 ± 0.19 ^d^	4.1 ± 0.17 ^f^
Methyl Jasmonate (ppm)	10	5.8 ± 0.17 ^c^	5.0 ± 0.13 ^d^	5.6 ± 0.16 ^c^	5.0 ± 0.14 ^e^
	20	5.9 ± 0.14 ^bc^	5.3 ± 0.16 ^cd^	5.7 ± 0.17 ^c^	5.1 ± 0.12 ^e^
	50	6.0 ± 0.19 ^b^	5.5 ± 0.14 ^c^	5.7 ± 0.14 ^c^	5.2 ± 0.17 ^de^
Melatonin (ppm)	10	5.9 ± 0.13 ^bc^	5.3 ± 0.16 ^cd^	5.8 ± 0.13 ^bc^	5.3 ± 0.14 ^d^
	20	6.0 ± 0.10 ^b^	5.5 ± 0.17 ^c^	5.8 ± 0.20 ^bc^	5.4 ± 0.15 ^cd^
	50	6.1 ± 0.15 ^ab^	5.7 ± 0.14 ^b^	5.9 ± 0.18 ^b^	5.5 ± 0.15 ^c^
Methyl Jasmonate + Melatonin (ppm)	10	6.0 ± 0.16 ^b^	5.6 ± 0.16 ^bc^	5.9 ± 0.15 ^b^	5.6 ± 0.18 ^bc^
	20	6.1 ± 0.18 ^ab^	5.9 ± 0.19 ^ab^	6.0 ± 0.17 ^ab^	5.7 ± 0.13 ^b^
	50	6.3 ± 0.20 ^a^	6.0 ± 0.20 ^a^	6.1 ± 0.21 ^a^	5.9 ± 0.20 ^a^

* In each column, values followed by the same letter are not significantly different according to Tukey’s HSD test (*p* ≤ 0.05); each value represents the mean of three replicates ± SD.

**Table 5:** Effects of foliar application of melatonin and/or methyl jasmonate solutions at different concentrations on the total soluble solids content (TSS, °Brix) in date palm fruits (cv. Barhi) stored at 4 °C for 28 days *.

Treatment		Days after Harvest					
		0	14	28	0	14	28
		2020			2021		
Control		27.8 ± 0.3 ^h^	29.5 ± 1.0 ^f^	31.8 ± 0.6 ^e^	28.0 ± 0.5 ^i^	29.8 ± 0.7 ^f^	31.9 ± 0.7 ^e^
Methyl Jasmonate (ppm)	10	28.7 ± 0.7 ^g^	30.7 ± 0.9 ^e^	32.8 ± 0.5 ^d^	28.9 ± 0.7 ^h^	30.8 ± 0.5 ^e^	32.8 ± 0.9 ^d^
	20	30.4 ± 1.0 ^f^	32.2 ± 1.1 ^d^	34.2 ± 0.9 ^c^	30.6 ± 0.8 ^g^	32.3 ± 0.6 ^d^	34.2 ± 0.7 ^c^
	50	31.7 ± 0.9 ^e^	33.5 ± 0.9 ^c^	35.1 ± 0.7 ^abc^	31.8 ± 0.6 ^f^	33.6 ± 0.6 ^c^	35.2 ± 0.9 ^bc^
Melatonin (ppm)	10	32.3 ± 0.6 ^d^	33.6 ± 0.7 ^bc^	34.5 ± 0.8 ^bc^	32.3 ± 0.8 ^e^	33.7 ± 0.7 ^c^	34.6 ± 1.0 ^c^
	20	32.8 ± 0.8 ^cd^	33.7 ± 0.5 ^bc^	34.6 ± 0.9 ^bc^	32.8 ± 0.9 ^de^	33.8 ± 1.1 ^c^	34.7 ± 0.8 ^c^
	50	33.1 ± 0.7 ^c^	34.2 ± 0.5 ^bc^	35.1 ± 1.0 ^bc^	33.2 ± 1.1 ^cd^	34.3 ± 1.0 ^bc^	35.2 ± 0.8 ^bc^
Methyl Jasmonate + Melatonin (ppm)	10	33.4 ± 1.0 ^bc^	34.6 ± 0.6 ^bc^	35.5 ± 0.7 ^b^	33.5 ± 1.0 ^bc^	34.6 ± 0.9 ^bc^	35.6 ± 0.6 ^b^
	20	33.8 ± 1.1 ^b^	34.7 ± 0.8 ^b^	35.3 ± 0.8 ^bc^	33.9 ± 0.7 ^b^	34.8 ± 0.8 ^b^	35.4 ± 0.9 ^bc^
	50	34.4 ± 0.9 ^a^	35.3 ± 0.9 ^a^	36.0 ± 0.9 ^a^	34.6 ± 1.0 ^a^	35.4 ± 1.0 ^a^	36.0 ± 0.8 ^a^

* In each column, values followed by the same letter are not significantly different according to Tukey’s HSD test (*p* ≤ 0.05); each value represents the mean of three replicates ± SD.

**Table 6:** Effects of foliar application of melatonin and/or methyl jasmonate solutions at different concentrations on the total sugars content (g/100 g fresh weight) in date palm fruits (cv. Barhi) stored at 4 °C for 28 days *.

Treatment		Days after Harvest					
		0	14	28	0	14	28
		2020			2021		
Control		24.4 ± 0.4 ^g^	26.4 ± 0.8 ^g^	25.5 ± 0.4 ^h^	24.4 ± 0.6 ^g^	26.5 ± 0.7 ^g^	25.4 ± 0.6 ^h^
Methyl Jasmonate (ppm)	10	25.7 ± 0.8 ^f^	27.3 ± 0.7 ^f^	26.4 ± 0.8 ^g^	25.7 ± 0.8 ^f^	27.3 ± 0.8 ^f^	26.5 ± 0.5 ^g^
	20	25.8 ± 0.7 ^f^	27.9 ± 0.6 ^f^	27.0 ± 0.5 ^g^	25.8 ± 0.9 ^f^	27.9 ± 0.5 ^ef^	27.1 ± 0.7 ^g^
	50	27.0 ± 0.9 ^e^	29.0 ± 0.9 ^e^	28.3 ± 0.6 ^f^	27.0 ± 0.7 ^e^	28.7 ± 0.5 ^e^	28.0 ± 0.6 ^f^
Melatonin (ppm)	10	28.7 ± 0.6 ^d^	30.6 ± 0.4 ^d^	29.7 ± 0.6 ^e^	28.7 ± 0.7 ^d^	30.7 ± 0.7 ^d^	29.7 ± 0.5 ^e^
	20	29.8 ± 1.0 ^c^	31.4 ± 0.8 ^c^	30.2 ± 1.0 ^de^	29.8 ± 0.9 ^c^	31.4 ± 1.0 ^cd^	30.3 ± 0.9 ^de^
	50	30.2 ± 1.0 ^bc^	32.1 ± 1.0 ^bc^	30.8 ± 0.9 ^cd^	30.2 ± 0.8 ^bc^	32.1 ± 0.7 ^bc^	30.8 ± 1.0 ^cd^
Methyl Jasmonate + Melatonin (ppm)	10	30.5 ± 0.8 ^bc^	32.4 ± 0.9 ^b^	31.4 ± 1.0 ^bc^	30.5 ± 0.7 ^bc^	32.4 ± 0.6 ^b^	31.5 ± 1.0 ^bc^
	20	30.9 ± 0.9 ^ab^	32.7 ± 1.1 ^b^	31.9 ± 1.0 ^ab^	30.9 ± 1.0 ^b^	32.7 ± 0.9 ^ab^	31.9 ± 0.9 ^ab^
	50	31.6 ± 1.1 ^a^	33.4 ± 1.0 ^a^	32.4 ± 0.9 ^a^	31.7 ± 1.0 ^a^	33.5 ± 0.9 ^a^	32.5 ± 1.1 ^a^

* In each column, values followed by the same letter are not significantly different according to Tukey’s HSD test (*p* ≤ 0.05); each value represents the mean of three replicates ± SD.

**Table 7:** Effects of foliar application of melatonin and/or methyl jasmonate solutions at different concentrations on the total acidity (g citric acid/100 mL juice) in date palm fruits (cv. Barhi) stored at 4 °C for 28 days *.

Treatment		Days after Harvest					
		0	14	28	0	14	28
		2020			2021		
Control		0.33 ± 0.07 ^a^	0.32 ± 0.06 ^a^	0.31 ± 0.06 ^a^	0.34 ± 0.05 ^a^	0.33 ± 0.08 ^a^	0.31 ± 0.07 ^a^
Methyl Jasmonate (ppm)	10	0.30 ± 0.03 ^b^	0.29 ± 0.05 ^b^	0.28 ± 0.07 ^b^	0.32 ± 0.06 ^b^	0.31 ± 0.05 ^b^	0.29 ± 0.06 ^b^
	20	0.28 ± 0.03 ^c^	0.26 ± 0.07 ^c^	0.26 ± 0.05 ^c^	0.30 ± 0.05 ^c^	0.29 ± 0.05 ^c^	0.27 ± 0.05 ^c^
	50	0.26 ± 0.05 ^c^	0.25 ± 0.06 ^c^	0.24 ± 0.05 ^d^	0.28 ± 0.07 ^d^	0.27 ± 0.06 ^d^	0.24 ± 0.07 ^d^
Melatonin (ppm)	10	0.23 ± 0.04 ^d^	0.21 ± 0.05 ^d^	0.20 ± 0.06 ^e^	0.25 ± 0.05 ^e^	0.24 ± 0.04 ^e^	0.21 ± 0.04 ^e^
	20	0.22 ± 0.06 ^de^	0.20 ± 0.04 ^de^	0.19 ± 0.05 ^e^	0.24 ± 0.06 ^ef^	0.23 ± 0.04 ^ef^	0.20 ± 0.04 ^e^
	50	0.21 ± 0.05 ^ef^	0.20 ± 0.06 ^de^	0.18 ± 0.06 ^ef^	0.22 ± 0.04 ^fg^	0.21 ± 0.05 ^fg^	0.20 ± 0.06 ^e^
Methyl Jasmonate + Melatonin (ppm)	10	0.20 ± 0.05 ^ef^	0.19 ± 0.05 ^ef^	0.17 ± 0.04 ^fg^	0.22 ± 0.05 ^fg^	0.20 ± 0.04 ^g^	0.19 ± 0.03 ^ef^
	20	0.19 ± 0.06 ^fg^	0.18 ± 0.04 ^fg^	0.17 ± 0.05 ^fg^	0.21 ± 0.05 ^gh^	0.19 ± 0.06 ^gh^	0.18 ± 0.04 ^fg^
	50	0.18 ± 0.07 ^g^	0.17 ± 0.04 ^g^	0.16 ± 0.04 ^g^	0.19 ± 0.03 ^h^	0.18 ± 0.05 ^h^	0.17 ± 0.04 ^g^

* In each column, values followed by the same letter are not significantly different according to Tukey’s HSD test (*p* ≤ 0.05); each value represents the mean of three replicates ± SD.

**Table 8:** Pearson moment correlations (r) matrix between fourteen variables recorded from date palm trees (cv. Barhi).

	RCC	N	P	K	YPP	BW	FL	FD	TSS	TS	TA	WL	FDC	FF
RCC	1													
N	0.93 ***	1												
P	0.98 ***	0.90 ***	1											
K	0.99 ***	0.95 ***	0.97 ***	1										
YPP	0.96 ***	0.98 ***	0.95 ***	0.97 ***	1									
BW	0.97 ***	0.94 ***	0.98 ***	0.97 ***	0.97 ***	1								
FL	0.99 ***	0.94 ***	0.97 ***	0.99 ***	0.97 ***	0.98 ***	1							
FD	0.98 ***	0.96 ***	0.95 ***	0.99 ***	0.98 ***	0.97 ***	0.99 ***	1						
TSS	0.75 ***	0.74 ***	0.81 ***	0.77 ***	0.80 ***	0.82 ***	0.76 ***	0.77 ***	1					
TS	0.97 ***	0.90 ***	0.98 ***	0.96 ***	0.94 ***	0.96 ***	0.97 ***	0.95 ***	0.83 ***	1				
TA	−0.93 ***	−0.86 ***	−0.96 ***	−0.91 ***	−0.91 ***	−0.95 ***	−0.93 ***	−0.91 ***	−0.87 ***	−0.97 ***	1			
WL	−0.38 **	−0.37 **	−0.37 **	−0.39 **	−0.39 **	−0.39 **	−0.37 **	−0.39 **	−0.39 **	−0.36 **	0.38 **	1		
FDC	−0.43 ***	−0.40 **	−0.45 ***	−0.43 ***	−0.43 ***	−0.44 ***	−0.42 ***	−0.43 ***	−0.44 ***	−0.43 ***	0.47 ***	0.87 ***	1	
FF	0.40 **	0.37 **	0.43 ***	0.39**	0.40 **	0.41 **	0.39 **	0.38 **	0.44 ***	0.42 ***	−0.47 ***	−0.84 ***	−0.95 ***	1

RCC = relative chlorophyll content, N = nitrogen content, P = phosphorus content, K = potassium content, YPP = yield/palm, BW = bunch weight, FL = fruit length, FD = fruit diameter, TSS = total soluble solids content, TS = total sugars, TA = total acidity, WL = fruit weight loss, FDC = fruit decay, and FF = fruit firmness. Values followed by ** or *** are significant at *p* ≤ 0.01 or *p* ≤ 0.001, respectively.

## Data Availability

The raw data supporting the conclusions of this article will be made available by the authors, without undue reservation.
